# Gaming in the Military: A Longitudinal Study of Changes in Gaming Behavior Among Conscripts During Military Service and Associated Risk Factors

**DOI:** 10.3389/fpsyt.2021.591038

**Published:** 2021-07-09

**Authors:** Olav Kjellevold Olsen, Ståle Pallesen, Helga Myrseth

**Affiliations:** ^1^Department of Psychosocial Science, University of Bergen, Bergen, Norway; ^2^Department of Leadership and Organizational Behaviour, BI Norwegian Business School, Oslo, Norway; ^3^Betanien hospital, Psychiatric Outpatient Clinic for Child and Adolescense, Skien, Norway

**Keywords:** problem gaming, gaming addiction, conscripts, prevalence, longitudinal, military, leadership

## Abstract

A central task in military leadership is to take care of one's followers, which presupposes knowledge about relevant risk factors. Very little research has focused on the risks of developing problematic gaming behavior during military service. The present study tries to bridge this gap by assessing prevalence rates and associated risk factors of problem gaming in a sample of Norwegian conscripts across two time-points: at the beginning and end of duty. The sample comprised 2,555 individuals aged 18–24 years. A total of 1,017 (39.8%) completed the questionnaire at Time 1, ~1 month after starting the military service. Respondents who completed the first wave, at enrollment, were invited to participate in wave two, after completing their service. At Time 2, 259 (25.5%) participants responded. The prevalence rates of gaming addiction were 0.5% at Time 1 and 4.6% at Time 2, while problem gaming use was reported by 4.8% of the sample at Time 1 and 8.1% of the sample at Time 2. Paired sample *t*-tests revealed an overall significant increase in the mean scores on the Gaming Addiction Scale from T1 (*M* = 0.86, *SD* = 1.35) to T2 (*M* = 1.31, *SD* = 2.14), *t* = −2.40, *p* < 0.05. According to the reliable change index, 17.1% of the sample showed a reliable negative change, whereas 8.3% exhibited a reliable positive change in gaming addiction scores. However, no psychological variables measured at T1 (loneliness, boredom proneness-Internal, boredom proneness-External, anxiety, depression, game addiction, and time spent gaming) were related to attrition (from T1 to T2), or worsening of game addiction, while a positive relationship was observed between boredom proneness-External and reduced gaming addiction from T1 to T2. In sum, we observed a tendency toward a negative change in gaming behaviors during military service which may complicate the soldiers' reintegration into civilian life after their service. More research is needed to assess potential gaming problems in the Military.

## Changes in Gaming Behavior During Military Service and Associated Risk Factors

In Norway, military conscription is mandatory for men and women at the age of 17–19 years. The conscripts selected for military service must then leave their family and social network to join the military for 1 year. Schei and Søgaard (1) suggested that the individual's social identity at this age may be pliant, and that young conscripts may easily be swayed by external pressure. They examined the impact of military service on conscripts' smoking behavior, and found that the military service had a negative influence on the smoking behavior of young Norwegian men.

Union representatives for the conscripts in the Norwegian Armed Forces have expressed concerns because many conscripts spend many hours every day playing computer games. Some have speculated that a lack of leisure activities for conscripts may contribute to more gaming and gambling during conscription (2). The Norwegian national helpline for gambling and gaming problems has also reported that a growing number of conscripts, and parents of conscripts, have contacted the helpline because of economic problems due to online gaming (2). If gaming problems are increasing during conscription, the service period may have undesirable consequences for the individual solider, for the military and for the society to which the individual returns to after service. Given that the ability to take care of one's followers is found to represent a vital element in successful leadership, increased gaming behavior may represent a significant challenge to military leadership (3). This is supported by a previous study, showing that nearly 5% of the Norwegian conscripts had symptoms of problem gaming when entering military service, despite rigorous screening procedures prior to conscription (4). In this cross-sectional study, lower psychosocial well-being (boredom, loneliness, depression, and anxiety) prior to being drafted was found to be associated with gaming problems, explaining nearly 7% of the variance in gaming addiction scores beyond weekly time spent gaming. Although a variety of previous studies have linked gaming addiction to elevated levels of boredom (5), loneliness (6–8), depression (9–11), and anxiety (9, 10, 12); longitudinal studies supporting the assumption that these pre-existing states promote pathological gaming is scarce. However, there is some longitudinal evidence, suggesting that loneliness is a predictor of subsequent gaming addiction (13, 14); but there is also evidence that pathological gaming predicts increased levels of depression and anxiety (15), as well as loneliness (13). Hence, a reciprocal relationship between these variables may exist (13, 15).

It is worth noting that despite the large number of conscripts and young service members around the world, and the wide range of negative consequences related to problematic gaming, such as a loss of control and social isolation, no studies have thus far focused on how gaming habits are influenced by the military service. As a result, longitudinal research investigating how levels of gaming addiction develop during conscription is warranted. Myrseth et al. (4) suggested two opposite developmental patterns. One may assume that the military training is so time-consuming and demanding that opportunities to engage in gaming behavior are decreased during conscription. Alternatively, one can imagine a pattern in which mandatory military service predisposes for the experience of isolation, boredom and loneliness. The soldiers are usually far away from home and their social network, and they are often exposed to a monotonous and somewhat meaningless service (16), which in turn may make them vulnerable to develop or maintain a gaming problem. The military has a special obligation to take care of the conscripts during service; consequently, research investigating whether mandatory military service exposes soldiers to social influences that may increase their vulnerability in terms of developing gaming addiction could subsequently have implications for the designing of effective caretaking programs for soldiers during service.

Hence, the purpose of the present study was to investigate the development of gaming behavior during conscription. More specifically, the present study aimed at tracking conscripts from the beginning to the end of service, and to (1) investigate if the entering of service increase the chance of developing problem gaming, and (2) to explore possible risk factors associated with problematic levels of gaming among conscripts, in particular whether initial levels of boredom, loneliness, anxiety, and depression at the entry of service can predict levels of gaming problem at the end of service.

## Methods

### Participants and Procedure

The initial sample consisted of 2,555 persons randomly selected from a pool of conscripts who started their military service in Norway between August 2013 and January 2014. The mean age of the total sample was 19.5 years (*SD* = 1.01), and 80.3% were male. The first wave of the questionnaire was distributed to the participants 1 month after starting their military service. Due to logistical reasons, ~50% of the sample was recruited via mail, whereas the other half of the sample was recruited on the military base. The respondents had the option of completing the questionnaire on paper or completing the questionnaire online. After 2 weeks, a reminder was sent to the recipients' e-mail addresses, after 4 weeks a reminder was sent to the participants' mobile phone with a web-link to the questionnaire, and after 7 weeks, a new postal reminder was sent to those who had not already returned the questionnaire. A total of 1,017 questionnaires were completed and returned, yielding a response rate of 39.8% in the first wave. The participants consisted of 817 men and 200 women. Participants who responded in the first wave were contacted again after completing their 1 year military service and asked to complete the questionnaire again. Due to a failure to locate addresses, 50 questionnaires were returned by the postal service. In the second wave, 258 of the 1,017 questionnaires were completed and returned, yielding a response rate of 26.7%. Similar to the first wave, the participants could choose to complete the questionnaire on paper or online, with one e-mail reminder and one postal reminder sent to each participant in the second wave. Among all the participants who completed the survey, six were randomly drawn to receive an iPad or an iPad mini.

### Measures

#### Gaming Addiction Scale

The GAS includes seven items, one for each of the seven criteria suggested as core components of video game addiction (salience, tolerance, mood modification, withdrawal, relapse, conflict, and problems) (17). Respondents are asked to indicate how often each problematic incident had occurred over the last 6 months on a five-point Likert scale [never (1), rarely, sometimes, often, or very often (6)]. The Cronbach's alpha for the GAS in the present study was 0.82. Lemmens et al. (17) suggested that the cutoff score for video game addiction can be set in different ways. According to the monothetic approach, respondents have to endorse all seven items (i.e., a score of 3 or higher on all seven items) to be classified as addicted. Following the polythetic approach, which is a less strict procedure, the respondents have to endorse at least four of the seven items in order to be categorized as a problem gamer.

#### Robert's UCLA Loneliness Scale

The RULS-8 is adapted from the 20-item UCLA Loneliness Scale and consists of eight items measuring loneliness, in which respondents indicate how well each statement fits according to four response categories (never, seldom, sometimes, and often) (18).

#### Boredom Proneness Scale—Short Form

The propensity to experience boredom was assessed using the BPS-SF, which consists of 12 items measuring boredom proneness on a seven-point Likert scale, where respondents indicate how well they agree (7) or disagree (1) with each statement (19). The BPS-SF consists of two factors: lack of internal stimulation and lack of external stimulation. The scoring of all items in the lack of internal stimulation subscale is reversed, so that high scores on both subscales indicate boredom; lack of internal stimulation (BPS-SF_I) or lack of external stimulation (BPS-SF_E). The BPS-SF was found to show an improved fit compared to the original BPS, and has been found to be invariant across gender (19).

#### Hopkins Symptom Checklist−5

The SCL-5 (20) is a short form of the Hopkins Check List [SCL-25; (21)], measuring symptoms of anxiety (SCL-5_A) and depression (SCL-5_D). The SCL-5 has demonstrated high correlations (*r* = 0.92) with the original version (20).

### Ethics

The study was conducted in accordance with the Declaration of Helsinki, and was approved by the Regional committee for medical and health research ethics. Informed consent procedure was applied, and it was emphasized that participation was voluntary; participants were also informed that they could withdraw from the study at any time without stating a reason.

### Statistical Analyses

The data were analyzed using the SPSS version 24 (22). For missing data, we used mean-substitution if the respondent had answered a minimum of 75% of the items in that particular subscale. Descriptive statistics were calculated in terms of distribution, and paired sample *t*-tests were conducted to investigate whether there were significant changes in the mean scores on gaming addiction (GAS), anxiety (SCL-5_A), and depression (SCL-5_D) from T1 to T2. In order to investigate whether attrition was related to the scores at T1 on loneliness (RULS-8), boredom proneness-I (BPS-SF_I), boredom proneness-E (BPS-SF_E), anxiety (SCL-5_A), depression (SCL-5_D), game addiction (GAS), and time spent gaming; these variables were regressed, using a logistic regression analysis, on participation at T2 (yes = 0, no = 1) which constituted the dependent variable.

A reliable change index (23, 24) was also calculated for gaming addiction scores from T1 to T2.

With the aim of identifying psychological factors at T1 that could predict changes in gaming problems from T1 to T2 the scores on loneliness, boredom proneness-I, boredom proneness-E, anxiety, and depression at T1 were included as independent variables in a multinomial regression analysis where no change from T1 to T2 on the gaming addiction scale constituted the reference group (52.8%), and where the other groups were those who improved (23.8%) and those who worsened (23.4%). A multinomial regression analysis was preferred over a linear regression analysis as the change scores on the gaming addiction between T1 and T2 were quite restricted and since most did not change. A similar approach was used for analyzing changes in time spent gaming from T1 to T2. No change (22.8%) comprised the reference group, whereas reduction (38.6%) and increase (38.6%) comprised the two other groups. The reason for limiting the number of groups is that the statistical power would be too small with a more nuanced categorization.

## Results

### Demographics

Demographic characteristics of the sample are reported in Table 1. The majority of the sample (77.3%) did not have a partner and had high school as the highest completed education (96%). Participants were distributed across all military branches, including Navy (34.7%), Army (29.1%), Air Force (27.8%), and King's Guard/Home Guard (8.3%). Most of them had participated in gaming during the last 6 months (84% at T1 and 79.1% at T2). The mean number of hours of weekly gaming were 11.6 at T1 and 10.4 at T2. More than three quarters had completed their military service at T2, while 0.4% had quit before the end of service due to gaming problems, and 22.1% had quit due to other reasons.

**Table 1:** Demographic characteristics of the sample.

**Variables**		**T1 (*****n*** **= 1,017)**		**T2 (*****n*** **= 259)**	
		**%**	**Mean (*SD*)**	**%**	**Mean (*SD*)**
Gender	Male	80.3		82.8	
	Female	19.7		17.2	
Age			19.45 (1.01)		
Marital status	Partner	22.7			
	Not partner	77.3			
Military division	Navy	34.7			
	Army	29.1			
	Airforce	27.8			
	King's Guard/Home Guard	8.3			
Education	Elementary school (10 years)	1.7			
	High school (13 years)	96.0			
	Lower University/college degree	2.0			
	Higher university/college degree	0.3			
Gaming	Participated in gaming last 6 months	84.0		79.1	
Weekly gaming	Number of hours		11.6 (13.6)		10.4 (10.3)
Problem gaming awareness	I think I play too much	5.9		5.9	
	I think I have problem with gaming	1.1		0	
	My partner is worried	4.1		1.5	
Service accomplishment	Completed service (>320 days)			77.5	
	Quit before end of service due to gaming problems			0.4	
	Quit before end of service due to other reasons			22.1	

### Participant Attrition From T1 to T2

None of the psychological variables (loneliness, boredom proneness-I, boredom proneness-E, anxiety, depression, game addiction, and time spent gaming) at T1 were related to attrition (from T1 to T2). The results are shown in Table 2. The overall regression model was not significant (χ^2^ = 5.6, *df* = 7, *p* = 0.59).

**Table 2:** Regression analysis results where loneliness, boredom proneness-I, boredom proneness-E, anxiety, depression, and game addiction scores at T1 are regressed on participation at T2 (yes = 0, no = 1).

**Variable**	**OR**	**95% CI for OR**	***p***
Loneliness	1.00	0.95–1.05	0.883
Boredom proneness-I	0.99	0.96–1.02	0.429
Boredom proneness-E	1.01	0.98–1.03	0.679
Anxiety	1.19	0.96–1.48	0.118
Depression	0.98	0.86–1.12	0.762
Game Addiction	0.96	0.84–1.10	0.542
Time spent gaming	1.01	1.00–1.02	0.214

*N = 979*.

*OR, odds ratio; 95% CI, 95% confidence interval; p, probability; Boredom proneness-I, boredom proneness—lack of internal stimulation; Boredom proneness-E, boredom proneness—lack of external stimulation*.

### Change in Gaming Behavior From Enrollment (T1) to End of service (T2)

At both T1 and T2, 5.9% of the conscripts responded that they thought they played too much, but only 1.1% admitted that they thought they had a gaming problem at T1 while none admitted to having a problem with gaming at T2 (see Table 1). At T1, 78.8% of the 1,017 respondents were classified as normal gamers, 4.8% as problem gamers, 0.5% as addicted gamers, and 15.9% as non-gamers (i.e., had not participated in gaming during the past 6 months). Of those who responded at T2 (*n* = 259), 65.6% were classified as normal gamers, 8.1% as problem gamers, 4.6% as addicted gamers, and 21.7% as non-gamers (see Table 3).

**Table 3:** Prevalence rates for gaming problems in the population of gamers and in the total sample.

	**T 1**		**T 2**	
	**Gaming sample**	**Total sample**	**Gaming sample**	**Total sample**
**Type of Gamer**	**(*n* = 855)**	**(*n* = 1,017)**	**(*n* = 206)**	**(*n* = 259)**
Non-gamer		15.9%		21.7%
Normal gamer	94.2%	78.8%	83.7%	65.6%
Problem gamer	5.2%	4.8%	10.3%	8.1%
Addicted gamer	0.6%	0.5%	5.9%	4.6%

Paired sample *t*-tests exhibited an overall significant increase in the mean total scores on gaming addiction from T1 (*M* = 0.86, *SD* = 1.35) to T2 (*M* = 1.31, *SD* = 2.14), *t* = −2.40, *p* < 0.05. In addition, paired sample *t*-tests showed an overall significant increase in the mean scores on Anxiety from T1 (*M* = 2.46, *SD* = 0.83) to T2 (*M* = 3.03, *SD* = 1.27), *t* = −6.51, *p* < 0.001, as well as a significant decrease in the mean scores on Depression from T1 (*M* = 3.99, *SD* = 1.45) to T2 (*M* = 3.01, *SD* = 1.38), *t* = 9.52, *p* < 0.001. Register data showed that only one of those identified as addicted gamers at T1 completed their military service.

#### Reliable Change Index

Change indexes for the total scores on the GAS for each individual were calculated (GAS-total_1_- GAS-total_2_). At T2, 41.4% of the sample showed no change on the total GAS score, whereas 30.9% showed deterioration and 27.7% showed improvement from T1. A reliable change index (RCI) was also calculated [RC = (x_2_-x_1_):S_diff_], and the RC criterion was 2.10, thereby indicating that every change >2.1 should be regarded as reliable (i.e., an increase or decrease of at least 3 points from the initial total GAS score). According to this criterion, 17.1% of the sample showed a clinical significant negative change (deterioration) in total GAS- scores at T2, while 8.3% showed a clinical significant positive change (improvement).

### Predicting Changes in Gaming Problem Symptoms From T1 to T2

Table 4 shows the result from the multinomial regression analysis outcome where factors assessed at T1 (loneliness, boredom proneness-I, boredom proneness-E, anxiety, and depression) were used as independent variables predicting worsening or improvement of gaming problems from T1 to T2. The final model was not significant [χ^2^ = 15.4, *df* = 10, *p* = 0.12, *R*^2^(Nagelkerke) = 0.086]. Still, boredom proneness-E at T1 was associated with improvement in gaming problems from T1 to T2.

**Table 4:** Multinomial regression analysis summary for loneliness, boredom proneness-I, boredom proneness-E, anxiety, and depression at T1 predicting worsening or improvement game addiction symptoms from T1 to T2 (significant findings are shown in bold).

	**Worsening**^**a**^			**Improvement**^**a**^		
	**OR**	**95% CI OR**	***p***	**OR**	**95% CI OR**	**p**
Loneliness	0.95	0.84–1.06	0.345	1.06	0.96–1.18	0.277
BPS-internal stimulation	1.04	0.97–1.11	0.254	1.01	0.94–1.08	0.854
BPS-external stimulation	1.05	0.99–1.11	0.121	**1.07**	**1.00–1.14**	**0.047**
Anxiety	1.17	0.71–1.94	0.532	1.11	0.70–1.76	0.652
Depression	0.91	0.68–1.22	0.515	1.07	0.83–1.38	0.616

a *No change comprised the reference category*.

*BPS, boredom proneness; OR, odds ratio; 95% CI, 95% confidence interval; p, probability*.

### Predicting Changes in Time Spent Gaming From T1 to T2

Table 5 shows the result from the multinomial regression analysis outcome where factors assessed at T1 (loneliness, boredom proneness-I, boredom proneness-E, anxiety, and depression) were used as independent variables predicting increase or reduction in time spent gaming from T1 to T2. The final model was not significant [χ^2^ = 16.1, *df* = 10, *p* = 0.10, *R*^2^(Nagelkerke) = 0.072]. Still, boredom proneness-I and boredom proneness-E at T1 was associated with increase in time spent gaming from T1 to T2.

**Table 5:** Multinomial regression analysis summary for loneliness, boredom proneness-I, boredom proneness-E, anxiety, and depression at T1 predicting increase or decrease in time spent gaming from T1 to T2 (significant findings are shown in bold).

	**Increase**^**a**^			**Decrease**^**a**^		
	**OR**	**95% CI OR**	***p***	**OR**	**95% CI OR**	***p***
Loneliness	0.92	0.82–1.04	0.168	0.91	0.81–1.02	0.112
BPS-internal stimulation	**1.10**	**1.02–1.18**	**0.018**	1.06	0.99–1.14	0.110
BPS-external stimulation	**1.10**	**1.03–1.18**	**0.006**	1.04	0.97–1.11	0.278
Anxiety	0.88	0.51–1.51	0.639	1.12	0.68–1.85	0.654
Depression	0.93	0.68–1.26	0.620	0.99	0.73–1.33	0.940

a *No change comprised the reference category*.

*BPS, boredom proneness; OR, odds ratio; 95% CI, 95% confidence interval; p, probability*.

## Discussion

The results from the present study showed that 5.3% of the 1,017 respondents were classified as a problem or addicted gamer at T1, whereas 12.7% of the respondents at T2 (*n* = 259) were classified as problem or addicted gamers. The most recent prevalence study of problem gaming in Norway found a prevalence of 3.3% of problem gaming among the general population (16–75 years), while 8.1% of the youngest age group (aged 16–25 years) were classified as a problem or addicted gamer (25). Hence, in our sample, it seems as if conscripts show higher levels of gaming problems at the end of their service compared to the similar age group in the general population. The fact that a greater proportion of the conscripts were classified as problem and addicted gamers at T2 compared to T1 may indicate a negative development of gaming problems and addiction during mandatory military service. The proportion of conscripts who reported having participated in gaming during the last 6 months were 84% at T1 and 79.1% at T2. Still, according to the reliable change index, more than 17% showed deterioration while only 8% showed improvement on the gaming addiction index. Additional research investigating a larger sample is needed in order to draw conclusion about the development of gaming behavior during mandatory military service.

It is worth noting that attrition in terms of participation across both T1 and T2 was found unrelated to all psychological variables included in the study (e.g., loneliness, boredom) as shown in Table 2. Thus, the relatively high attrition rate may be a result of service demands, like sailing in the navy or operating in the field as infantry soldiers, obstructing the distribution of surveys and subsequently the response rate. It is also worth noting that none of the conscripts defined as addicted gamers at T1 responded at T2. One may speculate if this is due to an increase in gaming problems during service, thereby causing a lack of adjustment to the service requirements and an early dismissal from service. Register data showed that only one of those identified as addicted gamer at T1 completed the military service. Future studies should therefore monitor to what degree gaming problems predict a lack of coping and adjustment to service requirements.

Because gaming addiction is usually characterized by a loss of control, distorted attention, and neglect of other activities (17), gaming addiction may be particularly problematic in a military context, in which task performance often requires optimal physical and mental functioning. The results from the present study may indicate that gaming and gaming addiction possibly increase during conscription, but future studies investigating this issue further are needed in order to draw firm conclusions regarding the development of gaming addiction among conscripts in general. If levels of gaming addiction increases during conscription, one may speculate whether organizational or structural circumstances of the conscription are contributing to this negative development. In the Norwegian Armed Forces, gaming facilities (Wifi, computers, games, Play Station etc.) are usually provided for conscripts as a part of their welfare opportunities. Since levels of gaming problems have previously been found to be related to the availability of gaming (26, 27), one might assume that the availability of gaming opportunities may contribute to a negative development of gaming behavior among the soldiers. However, the present study found that even though the propensity to experience boredom—both externally and internally—at T1 predicts more time spent gaming at T2, a negative effect in terms of increased gaming addiction symptoms at T2 cannot be found for these people. Thus, no relationship between how much you play and level of problems can be found. This may indicate that ability to control your gaming (e.g., stop when you want to) is an important factor.

Furthermore, it could be expected that lack of stimulating service experiences may cause the soldiers to seek out other sources of stimulation to maintain a sense of motivation, as claimed by several critiques [e.g., (16)]. Military boredom is hardly a new phenomenon, as war has previously been described as consisting of 5% horror and 95% boredom (28). Thus, a failure to cope effectively with boredom during conscription may result in negative behaviors such as gaming addiction. However, the present study found that soldiers that have a high need for external stimulation in order to avoid a sense of boredom, measured at T1, actually experience a significant improvement in symptoms of gaming addiction at the end of service (T2)—and no worsening. This indicates that military service in general may represent a more stimulating and exciting experience than suggested in the literature [e.g., (28)], functioning as a buffer toward gaming addiction even for soldiers that get easily bored.

Previous studies have also reported associations between elevated levels of depression (9–11) and elevated levels of anxiety (9, 10, 12) and gaming problems. However, the results of the present study showed that psychological variables in terms of loneliness, anxiety, and depression at T1 did not explain any improvement or worsening of symptoms of gaming addiction. One possible explanation for this, is that gaming may also represent something positive for the soldier, e.g., entertainment. Gaming has previously been linked to coping motivation and social motivation (29), so it is possible to imagine that problem gamers cope with (or escape from) real-life problems through gaming, and that their gaming fulfills their social needs, hence making them less prone for symptoms of depression or loneliness. Studies have reported social and psychological benefits from gaming (30–33), thus indicating that the relationship between gaming and psychosocial health is complex. Furthermore, this may also be a result of a rather rigorous selection process focusing on mental health, resulting in a population of serving soldiers of generally good mental health and functioning, reducing the risk of developing addiction. The lack of relationship between these intrapsychological variables and increase in gaming addiction symptoms may also be seen as a result of high social support and group cohesion usually found in military groups (28), which function as a buffer between individual vulnerabilities and development of gaming problems.

At the moment, there is no single conceptual theoretical model of gaming addiction that explain the development of gaming addiction, but research the development of gambling addiction may shed some light on this issue. According to (34) pathways model of the development of gambling addiction, there are multiple biological, psychological and ecological variables accounting for the development of pathological gambling. They propose a pathways model that integrates the complex array of biological, personality, developmental, cognitive, learning theory, and ecological determinants that may account for the development of pathological gambling, and proposed that there are three distinct subgroups of gamblers; the behaviorally conditioned problem gamblers, the emotionally vulnerable problem gamblers, and the antisocial, impulsivist problem gamblers. If we apply this theoretical framework to the present study, and assume that the development of gaming addiction follow similar sequences/pathways as the development of gambling addiction, it would be reasonable to assume that the latter two subtypes (the emotionally vulnerable gamers and the antisocial impulsivist gamers) will not be represented in the present study due to rigorous selection procedures where ~40% are discharged from duty in the Norwegian Military Forces. According to this model, the behaviorally conditioned gamers are not characterized by any specific premorbid feature of psychopathology. Rather, the availability and accessibility of opportunities to participate in gaming, in addition to classical and operant conditioning factors of the game itself leading to increased participation and the development of habitual patterns of gaming and cognitive processes resulting in faulty beliefs related to personal skills and winning contribute to the development of addition for this group.

### Practical Implications

Because addiction is likely to have a negative impact on the training, operative ability, and task performance of the soldiers, it has previously been suggested to use gaming addiction as an exclusion criteria for drafting (4). However, the fact that more than 17% showed a reliable deterioration of gaming addiction during conscription, and the fact that 13.5% of those who were characterized as normal gamers at the beginning of service were categorized as problem or addicted gamers at the end of service, indicates that merely revising the selection criteria may not be sufficient. Further action from military leaders may be required in order to cope with potential gaming problems.

Since levels of gaming problems have previously been found to be related to availability of gaming (26, 27), introducing restrictions for gaming possibilities may consequently potentially contribute to reducing gaming problems during conscription. Furthermore, developing and stimulating alternative leisure activities may also have a positive impact, and could possibly counteract a negative development of gaming problems. In addition, the qualitative aspects of conscripts' experiences could be monitored more closely during service, particularly in terms of boredom, loneliness, anxiety, and depression, in order to develop strategies to protect the more vulnerable groups of conscripts. Lastly, implementing screening in terms of gaming problems at the end of service could also be useful in order to offer coaching to potential problem gamers and possibly follow-ups after dismissal. This may further help to reduce a spillover from potential gaming problems developed during service into civilian life post-service.

### Limitations of the Present Study and Future Directions

The high dropout rates and relatively small sample size at T2 represent a potential threat to the validity of the statistical conclusion; consequently, future studies with larger samples are needed in order to investigate the potential predictors of gaming problems at the end of conscription. As only 26% of the respondents at T1 responded at T2, we may question whether they are representative of the whole group of conscripts. However, the analysis showed that there were no significant differences at T1 between those who responded at T2 and those who did not respond on neither severity of gaming addiction, weekly time spent gaming nor on any of the psychosocial factors. However, studies have shown that non-response is usually associated with pathology and there is more likely an underreporting of prevalence rates (35). Hence, it seems unlikely that there is less gaming problems among those who did not respond at T2, which supports our conclusions of a negative development of gaming problems during conscription.

The results would also have been strengthened if we had been able to control for or account for possible effects of concomitant treatments that conscripts may have received during their services, such as psychotherapy or medications, which might have effected levels of depression, anxiety, or additive behaviors at the end of service/T2. However, such information was not available due to confidentially regulations in the Norwegian Military forces.

Future studies should also include more time-points (not only pre-post-measures) to help investigate possible fluctuations of gaming addiction throughout a conscript's service. In order to identify possible predictors of gaming problems post-service, future studies may need to include variables other than those included in the present study, e.g., control for the availability of gaming facilities.

## Conclusions

In our sample of conscripts, the proportion of problem and addicted gamers was higher at the end of service (8.1 and 4.6% for problem and addicted gamers, respectively) compared to pre-conscription rates (5.2 and 0.6% for problem and addicted gamers, respectively). Although 8.3% of the conscripts showed a reliable improvement in gaming problems during service, there seems to be a negative development of more gaming problems during military service for 17% of the conscripts and higher prevalence rates of gaming problems were found among conscripts at the end of service compared to the general population. More studies are needed to investigate this issue further.

## Data Availability Statement

The raw data supporting the conclusions of this article will be made available by the authors, without undue reservation.

## Ethics Statement

The studies involving human participants were reviewed and approved by Norwegian Regional committee for medical and health research ethics (rec west). The patients/participants provided their written informed consent to participate in this study.

## Author Contributions

All authors contributed to the planning, data collection, theory development, data analysis and discussion.

## Conflict of Interest

The authors declare that the research was conducted in the absence of any commercial or financial relationships that could be construed as a potential conflict of interest.
